# Extracting Optimal Number of Features for Machine Learning Models in Multilayer IoT Attacks

**DOI:** 10.3390/s24248121

**Published:** 2024-12-19

**Authors:** Badeea Al Sukhni, Soumya K. Manna, Jugal M. Dave, Leishi Zhang

**Affiliations:** 1 School of Engineering, Technology and Design, Canterbury Christ Church University, Canterbury CT1 1QU, UK; leishi.zhang@canterbury.ac.uk; 2 Directorate of Research and Publications, Rashtriya Raksha University, Gandhinagar 382305, India; jugal.dave@rru.ac.in

**Keywords:** IoT attacks, multilayer security, feature selection, feature weighting, machine learning, human–machine teaming

## Abstract

The rapid integration of Internet of Things (IoT) systems in various sectors has escalated security risks due to sophisticated multilayer attacks that compromise multiple security layers and lead to significant data loss, personal information theft, financial losses etc. Existing research on multilayer IoT attacks exhibits gaps in real-world applicability, due to reliance on outdated datasets with a limited focus on adaptive, dynamic approaches to address multilayer vulnerabilities. Additionally, the complete reliance on automated processes without integrating human expertise in feature selection and weighting processes may affect the reliability of detection models. Therefore, this research aims to develop a Semi-Automated Intrusion Detection System (SAIDS) that integrates efficient feature selection, feature weighting, normalisation, visualisation, and human–machine interaction to detect and identify multilayer attacks, enhancing mitigation strategies. The proposed framework managed to extract an optimal set of 13 significant features out of 64 in the Edge-IIoT dataset, which is crucial for the efficient detection and classification of multilayer attacks, and also outperforms the performance of the KNN model compared to other classifiers in binary classification. The KNN algorithm demonstrated an average accuracy exceeding 94% in detecting several multilayer attacks such as UDP, ICMP, HTTP flood, MITM, TCP SYN, XSS, SQL injection, etc.

## 1. Introduction

The rise of the Internet of Things has changed how we live and work, leading to new developments in areas such as health, education, energy, and transportation. This change is driven by the increasing use of IoT devices, such as smart metres, wearable technology, and smartphones. The number of these devices is expected to reach 55.7 billion by 2025, according to the International Data Corporation (Global market intelligence, data, and events provider, Massachusetts, United States) [1]. However, this growth also brings new challenges, especially in terms of security and privacy. This is because IoT devices are often designed with limited computational resources and processing power, making them easy targets for cyber attackers. Estimations from the National Cyber Security Centre (Government Cyber Security Organization, London, United Kingdom) suggest that around 98% of IoT traffic remains unencrypted [2].

Furthermore, the architecture of the IoT introduces specific vulnerabilities. Its heterogeneous and interconnected structure, consisting of multiple layers and diverse components, increases susceptibility to attacks. Each layer of the IoT framework, from the perception layer that directly interacts with the physical world to the application layer that provides services to users, is exposed to unique vulnerabilities. For example, the communication layer, responsible for the exchange of data between devices, stands as a prime target for Man-In-The-Middle (MITM) attacks. Similarly, the application layer, which interacts with end-users, often becomes a significant target for exploitation due to software vulnerabilities.

Single-layer attacks, such as physical layer jamming, typically target the physical layer in the IoT architecture, whereas the network layer is disrupted by radio frequency interference. These attacks can reduce system performance but often remain isolated to the affected layers, without affecting the overall integrity of the system. In contrast, multilayer attacks, such as a combination of side-channel and MITM attacks, exploit vulnerabilities across multiple layers of the IoT system. These attacks compound the risks by targeting not only the network layer but also the application layer and physical layer, where data interception can occur. By affecting multiple layers, multilayer attacks can compromise the entire IoT ecosystem’s security, leading to more significant threats like data loss and personal information theft. Therefore, multilayer attacks are inherently more dangerous than single-layer attacks due to their ability to affect multiple components of the IoT infrastructure simultaneously, making them more difficult to detect and mitigate. According to the IBM Security X-Force report for 2022, 74% of IoT attacks are caused by Mozi botnets launching MITM attacks [3]. Also, according to the National Cyber Security Centre, UK, in 2020, a Russian hacking group leaked documents about a project aiming to create an IoT botnet inspired by the Mirai botnet, targeting security cameras and network video recorders to perform password attacks and grow the botnet. This botnet, once large enough, could launch powerful DDoS attacks, illustrating the significant threat of IoT vulnerabilities being exploited by both state and non-state actors [4]. This research specifically examines the risks posed by multilayer attacks and explores detection mechanisms for these complex threats.

In the landscape of IoT security, the concept of multilayer attacks has increasingly been recognised as a critical area of concern, as addressed by [5,6]. The authors in [5] identify types of multilayer attacks like DoS, side-channel, MITM, and cryptanalysis, whereas the authors in [6] focus on RFID security, including attacks like covert channels, side-channel attacks, replay attacks, and traffic analysis in their definition of multilayer attacks. Alongside these, refs. [7,8] have also explored the categories of multilayer attacks such as side-channel, cryptoanalysis, and MITM attacks. These studies show that multilayer security threats in IoT systems are becoming more complex. Hence, this makes it necessary to have secure measures to protect against different types of multilayer attacks, and this can be achieved by understanding the comprehensive taxonomy of multilayer attacks.

**Figure 1:**
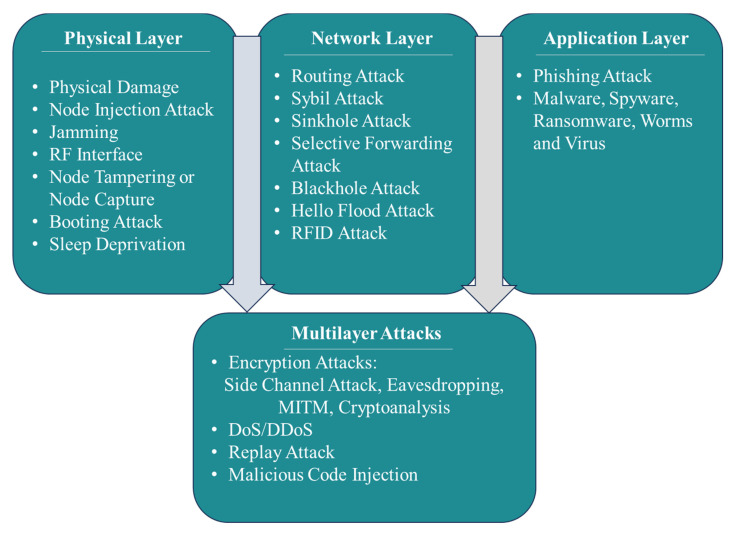
Taxonomy of multilayer IoT attacks.

Figure 1 provides a comprehensive taxonomy of multilayer attacks within IoT systems, distinguishing them from the single-layer attacks associated with the physical, network, and application layers, and a specialised focus on the nature of multilayer attacks that span across these layers. The physical layer is where direct hardware damage or disrupting physical operations occur through means like jamming and node tampering. The network layer encounters threats that interrupt the routing of data, including routing and sybil attacks, which affect the network’s integrity. Application layer attacks aim at the user interface, deploying malware and phishing tactics to exploit software vulnerabilities.

From our primary investigation, it appears that multilayer IoT attacks mainly consist of Encryption Attacks, DoS/DDoS attacks, replay attacks, and Malicious Code Injection Attacks [9,10]. Encryption Attacks refer to a group of attacks that compromise the IoT’s encryption methods with techniques like side channelling, cryptanalysis, and MITM attacks. Side Channel Attacks exploit encryption processes across IoT layers, while MITM attacks intercept communications to control network traffic. Cryptanalysis Attacks involve attempts to decrypt messages without the encryption key, and Eavesdropping Attacks capture data via insecure channels, compromising privacy and confidentiality.

DDoS attacks aim to flood servers with overwhelming traffic, these attacks disrupt services, as seen in ICMP, UDP, HTTP, and SYN flooding. These attacks often involve botnets, a network of compromised devices, posing a severe threat to IoT infrastructure. Replay attacks particularly impact the physical and network layers, where attackers rebroadcast valid communication to gain unauthorised access or disrupt services. Code Injection Attacks like SQL injection and Cross-Site Scripting (XSS) inject malicious code into IoT nodes, impacting both physical and application layers, and posing a threat to system functionality to gain unauthorised access.

## 2. Literature Review

Several techniques have been developed so far for detecting and mitigating multilayer attacks in wireless multi-hop networks. Refs. [9,10,11] have focused on detecting attacks such as packet dropping, route misdirection, DDoS, and MITM attacks, with approaches ranging from cross-layer interactions to behaviour-based anomaly detection techniques. The authors in [12] have advanced these techniques by combining device-driver packet filters and remote firewalls to mitigate DDoS attacks. A study conducted by the authors in [13] used a distributed mobile agents approach to detect multilayer packet-dropping attacks in mobile ad hoc networks (MANET) and used NSv2 software to implement their approach.

Alongside other traditional approaches, the application of machine learning (ML) in detecting IoT attacks has been evidenced by researchers. For example, the authors in [14] proposed a machine learning-based system for detecting DDoS attacks in IoT environments, employing supervised learning models such as Decision Trees (DT) and manual feature selection based on the attack type. Their work demonstrates the potential of machine learning algorithms in accurately detecting various DDoS attacks with an accuracy of around 97% in IoT networks. The authors of [15] proposed a semi-supervised machine learning model named Learning-Driven Detection Mitigation (LEDEM), designed to detect DDoS attacks in IoT networks by leveraging cloud and SDN technologies. Their model identifies DDoS attacks with an accuracy rate of 96.28%. A study conducted by the authors in [16] introduced a hybrid machine learning system that combines Random Forest (RF) for feature selection with Classification and Regression Trees (CART) for classifying various types of IoT network attacks, such as wormholes, shellcodes, DoS, and backdoors. Tested on the UNSW-NB15 dataset, their approach achieved an accuracy of 95.37%. The authors in [17] proposed a framework based on the NSL-KDD dataset to detect cyber-attacks such as Probe, U2R, R2L, and DDoS. The authors demonstrated the effectiveness of their framework through experiments, including a real smart building scenario, achieving a detection accuracy of anomalies at 99.71% using one-class SVM. Although the ML models have achieved high success in detecting one type of multilayer attack, which is the DoS attack, they face challenges in identifying different attack types. Also, the author’s use of an outdated dataset (the NSL-KDD) makes their models less suitable for modern IoT networks.

The effectiveness of the proposed solutions for securing MANET is demonstrated using NS2 simulations. However, real-world IoT environments may present unforeseen challenges not captured by simulation tools, such as interference and more complex attack scenarios. Behaviour-based systems often require significant time and computational resources to continuously update training data and may not quickly adapt to new or evolving attack patterns, which are common in IoT environments.

Furthermore, remote firewalls and packet filters primarily focus on network-layer attacks and overlook other dimensions of multilayer attacks, such as those targeting the application or perception layers. These approaches are also insufficient to protect against internal threats that have already bypassed the network perimeter. The distributed nature of mobile agents might introduce latency in detection, which is critical for real-time responses in IoT systems.

### 2.1. Feature Selection

By investigating feature selection methods, we aim to reduce the computational process for training machine learning models with minimum features by filtering out noise in the data and focusing on the most relevant information. Researchers have explored different wrapper and filter feature selection methods, such as the bijective soft set technique, correlation, fast-based-correlation feature (FCBF) algorithm, Information Gain (IG), and Gain Ratio (GR), which have proven beneficial in accurately identifying botnet, DoS, DDoS, and MITM attacks in IoT networks.

For example, ref. [18] focused on detecting botnet attacks in IoT networks using a wrapper feature selection method called the bijective soft set technique, introducing a novel metric named CorrACC. Their approach analysed the use of machine learning classifiers on the Bot-IoT dataset, and was proven to be successful with Decision Trees and Random Forest classifiers, achieving over 95% accuracy with the selection of seven key features. Further, ref. [19], applied Gain Ratio and Information Gain to detect DoS and DDoS attacks, reaching an impressive 99% accuracy and detection rate using 16 features on the BoT-IoT dataset and 19 features on the KDD Cup 1999 dataset. Similarly, ref. [20] assessed multiple machine learning algorithms to predict MITM, DoS, and scan attacks, using a correlation technique with a threshold of 0.6 for feature selection that yielded high accuracy detection using Decision Trees, and high Area Under the Curve (AUC) scores using Random Forests. Additionally, ref. [21] introduced a feature selection approach using Gain Ratio and Information Gain, coupled with mathematical techniques like intersection and union rules, to extract the most relevant features. Their method effectively identified relevant features for detecting DDoS and DoS attacks, resulting in high accuracy rates of nearly 99.98%.

Although these existing feature selection methods can extract significant features from datasets, it remains unclear which is the most effective. Also, each feature selection method has its pros and cons; even the accuracy of those methods invariably depends on the training dataset. Our primary aim is to ascertain the optimal number of significant features, irrespective of all feature selection methods used, by incorporating multiple feature selection methods in the decision-making process.

### 2.2. Feature Weighting

To scrutinise significant features through feature selection methods, employing feature weighting proves valuable when assigning scores/weights to each feature, indicating its significance in detecting IoT attacks within a dataset. Research has been carried out to develop advanced feature weighting methods for phishing site detection in smart cities. Ref. [22] presented a novel feature weighting method using hybrid bio-inspired algorithms, specifically Gray Wolf Optimization (GWO) and the Firefly Algorithm (FF). This technique significantly enhances the performance of an ANN used for classification, demonstrating a detection accuracy of 95.75%. In contrast, the Particle Swarm Optimization (PSO)-based feature weighting method developed by [23] achieved 95% accuracy during training and 93% accuracy in detection during testing, along with an impressive 98.4% accuracy rate in locating untrustworthy sites. Both studies underscore the effectiveness of employing feature weighting techniques in the domain of cybersecurity, particularly in the detection of phishing sites in smart city environments. Complementing these studies, ref. [24] focuses on IoT device security, employing a statistical aggregation (SA) and multi-objective optimisation method (based on the ratio analysis) for feature weighting of security authentication features. This method demonstrates a substantial accuracy improvement of 70% using the SA method. Moreover, the study conducted by [25] explains a combination of rule-based techniques and Multi-Objective Particle Swarm Optimization for feature selection. They also enhance attack detection in IoT-based wireless sensor networks by employing an advanced Multiclass Support Vector Machines classifier. To validate the effectiveness of their approach, the authors conducted experiments using the KDD ’99 Cup and CIDD datasets that showcase that their methodology not only enhances intruder detection accuracy but also effectively reduces false-positive rates.

In the existing state-of-the-art literature, studies focused on developing frameworks that incorporated machine learning models for single-layer attack identification and have demonstrated excellent performance [14,16,17]. However, to the authors’ knowledge, there is no comprehensive framework for leveraging machine learning models for detecting multilayer attacks in a unified approach that both detects the occurrence of multilayer IoT attack and classifies the type. Also, there is a gap in the research regarding feature selection and weighting methods across diverse IoT environments and attack vectors, especially for multilayer attacks. Also, there is a notable absence of discussion on incorporating human expertise in the loop of feature selection and weighting processes, which could enhance the interpretability and reliability of the detection models, especially in complex scenarios where automated methods might struggle. Building on our established taxonomy of multilayer IoT attacks as explained in our prior research [26,27], this novel Semi-Automated Intrusion Detection System (SAIDS) aimed to detect and identify multilayer attacks in IoT infrastructure. This approach compensates for the limitations of existing research that mostly focuses on single-layer attacks while offering a comprehensive and innovative approach to IoT multilayer attack detection methods:

1. The proposed approach comprises an ensemble feature analysis technique by combining multiple feature selection and feature weighting methods to identify the optimal number of significant features from IoT datasets.
2. The SAIDS approach includes Human–Machine Teaming by utilising human expertise in extracting feature selection and the tuning of data mining parameters to facilitate a more robust approach to identifying attack patterns to enhance the system’s adaptability and accuracy in detecting multilayer intrusions.
3. The SAIDS includes a unique visualisation tool that graphically represents how individual features influence the detection process to guide researchers and developers in understanding and selecting the minimum significant features.
4. The SAIDS employs a two-stage classification process where the first stage uses a binary classifier to filter traffic into normal and abnormal categories whereas the second stage applies multiclass classification to identify specific types of multilayer attacks, enabling targeted mitigation strategies.

The rest of this paper is organised as follows: Section 3 describes the proposed framework of the semi-automated tool. Section 4 discusses the implementation of the proposed approach SAIDS using the Edge-IIoTset Dataset. Section 5 presents the results of our experiments and further analysis. Section 6 concludes the findings and discusses possible areas for future research.

## 3. Methodology

**Figure 2:**
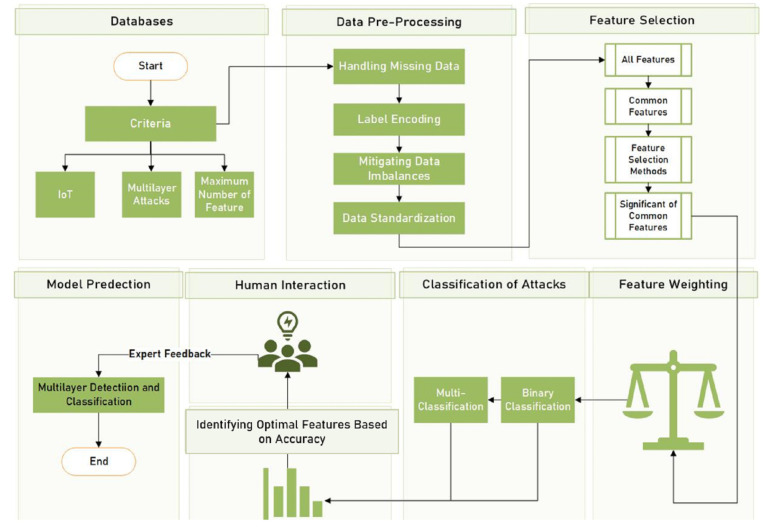
Semi-Automated Intrusion Detection System (SAIDS).

This section discusses the procedural methodology of the proposed framework, which incorporates feature selection, feature weighting, and a semi-automated approach where human expertise and machine learning algorithms work together, as illustrated in Figure 2. This collaboration ensures that cybersecurity experts thoroughly analyse the output of the semi-automated tool and provide essential feedback.

A. Datasets Selection

The methodology starts with selecting data from various sources while considering specific criteria. These criteria include IoT-specific datasets, datasets related to multilayer attacks, and considerations for the maximum number of features that can be processed by ML algorithms.

B. Data Pre-Processing

Pre-processing includes handling missing data and converting categorical data into a numerical format understandable by ML algorithms through label encoding. It includes scaling data on a common scale across all features. Also, it involves mitigating data imbalances to avoid bias towards a particular class, which is common in cybersecurity where attacks are rarer than normal events.

C. Feature Selection

This process begins with the identification of features common to multilayer attacks. Subsequently, various feature selection methods are applied to determine the most significant of common features. This is because irrelevant or redundant features may increase computational complexity, and sometimes negatively impact the model’s performance.

D. Feature Weighting

During this stage, weights are assigned to each of the selected features, aiding the machine learning algorithms in prioritising the most significant features throughout the learning process.

E. Machine Learning for Feature Selection

Machine Learning models were used to help with identifying the most important features for both classification tasks, namely, binary and multiclass classifications.

F. Identifying Optimal Features Based on Accuracy

A semi-automated tool is created for visualising the impact of sequentially adding top-weighted features into ML classifiers. This tool aids in guiding the selection of the most significant features that contribute to a higher accuracy rate. The visualisations assist the feature selection for both binary classification and multiclass classification.

G. Human Interaction

The inclusion of human expertise in the SAIDS framework is crucial, firstly, for understanding the features and converting the semi-structured IoT data into structured data. Additionally, cybersecurity experts interact with the semi-automated tool by selecting the minimum number of significant features, which reduces the computational power required and the detection time for multilayer attacks. Experts may also modify accuracy thresholds or intervene in cases where the model encounters rare attack types that require specialised knowledge. For instance, experts might adjust the weighting for underrepresented attack types that the model misclassifies due to their rarity, thereby enhancing detection in less common scenarios.

H. Models Predictions

The proposed framework incorporates two classification tasks: binary classification to distinguish between normal IoT traffic or malicious attacks, and multi-classification, for predicting multiple types of IoT attacks. If the IoT traffic is flagged as malicious multilayer attacks, the system further investigates to identify the type of multilayer attack through multiclass classification. Also, the system is designed to easily integrate and add classifiers as needed. For the specific use case in this study, we utilised classifiers such as Decision Tree, K-Nearest Neighbors, Naive Bayes, Random Forest, and Artificial Neural Networks as they are suitable for this prototype.

## 4. Implementation of the Proposed Approach (SAIDS) for Multilayer IoT Attack Detection Using the Edge-IIoTset Dataset

**Figure 3:**
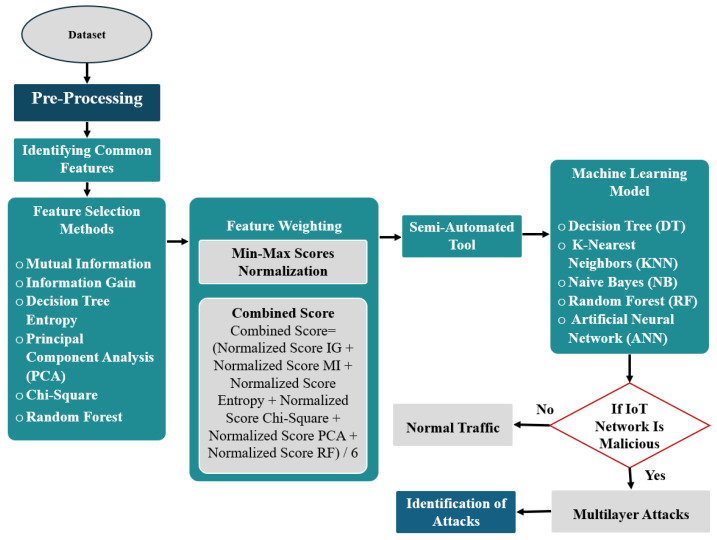
Implementation of SAIDS to Edge-IIoTset Dataset.

The proposed methodology is implemented utilising the Edge-IIoTset dataset, as described in Figure 3.

A. Dataset:

In this research, the latest and most comprehensive benchmark IoT cybersecurity dataset, the Edge-IIoTset dataset, is utilised [28]. While previous studies have employed this dataset to detect intrusions in IoT and industrial IoT systems [28,29,30,31,32,33,34] the detection and classification of multilayer attacks is limited and not explored. Instead, most studies have explored manual feature selection methods, with only one exception employing feature selection. Additionally, a limited number of studies have investigated the tuning of the hyperparameters of the models used.

The Edge-IIoTset dataset is particularly well-suited for multilayer attack detection due to its diverse range of attack types and comprehensive feature set. It contains data from 14 distinct attack types, including DDoS, MITM, injection, and malware attacks, which often span multiple layers of IoT architecture. This diversity allows SAIDS to evaluate multilayer attacks by analysing cross-layer interactions and patterns associated with these attacks. Furthermore, the dataset, generated from over 10 types of IoT devices, including sensors and detectors, provides a realistic representation of heterogeneous IoT environments where multiple device types are interconnected. The dataset’s 63 unique features capture a broad range of attributes, from network and transport layer details to application-level information, making it ideal for examining how multilayer attacks exploit vulnerabilities across different layers in the IoT stack.

The following pseudocode explains the Implementation of the SAIDS to the Edge-IIoTset Dataset in more detail. For each feature selection method, F_ranked[fs_method] holds a list of features, ordered/ranked by their scores in descending order of importance based on that specific feature selection method. Meanwhile, F_sorted is the final list of features, sorted by their combined scores after averaging the scores across all selection methods to assign a weight to each feature (See Algorithm). **Algorithm: SAIDS****Input:**Dataset D
Feature Set F = {f1, f2, …, fM}
Top Features to Select T**Output:**Optimal Features F_opt
Best ML Model M_best**Begin:****Step 1:** Preprocess (D)
**Step 2:** F_common = IdentifyCommonFeatures(D)
**Step 3:** Initialise containers:
     F_ranked = {}
     F_normalized = {}
     Scores_Sum = {}
     F_combined = {}
**Step 4:** Feature Selection and Normalisation:
     **for** each fs_method in [MI, IG, DTE, Chi^2^, PCA, RF]:
     F_scores = FeatureSelection (D, F_common, method=fs_method)
     F_ranked[fs_method] = SortFeatures (F_scores, descending=True)
     F_normalised[fs_method] = Normalise(F_scores)
          **for** each feature in F_normalised[fs_method]:
                                   **if** feature not in Scores_Sum:
                                        Scores_Sum[feature] = F_normalised[fs_method] [feature]
                                   **else:**

Scores_Sum[feature] += F_normalised[fs_method] [feature]
F_weighted = F_sorted
**Step 5:** Calculate Combined Scores:
     **for** feature in Scores_Sum:
         F_combined[feature] = Scores_Sum[feature] / len(F_normalised)
**Step 6:** Sort Features by Combined Scores:
         F_sorted = SortFeatures (F_combined, descending=True)
**Step 7:** Model Training and Selection:
         M_best_acc = 0
         F_opt = []
         M_best = None
         **for** N in range (1, T+1):
           F_subset = F_weighted [: N]
           **for** M in [DT, KNN, NB, RF, ANN]:
     M_tuned = HyperparameterOptimisation (M, F_subset)
     M_trained = Train (M_tuned, F_subset)
     M_metrics = Test (M_trained, F_subset)
     **if** M_metrics[’accuracy’] > M_best_acc:
M_best_acc = M_metrics[’accuracy’]
M_best = M_trained
F_opt = F_subset
    
**end if**

**end for**
         
**end for**

**Step 8:** VisualiseImpact(F_opt)
**Step 9:** HumanExpertReview (F_opt, M_best)
**Step 10:** FinaliseModel (M_best, F_opt)
     Return (F_opt, M_best)**End**

**Figure 4:**
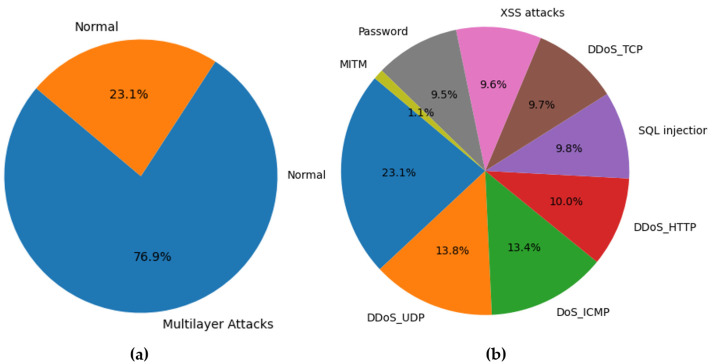
Distribution of Traffic: (**a**) normal and multilayer attacks, (**b**) normal and attack types.

B. Pre-Processing:

This stage focuses on preparing the “Edge-IIoTset” dataset for analysis by removing any missing or duplicate data. Also, we split the “frametime” feature into two separate attributes: “frame.time_WithoutIP” and “frame.time_WithIP”. This division is essential because the original attribute has both IP addresses and timestamps. This modification increases the total count of features in the dataset from 63 to 64. Out of the 14 different types of attacks present in the datasets, we chose to focus on multilayer-related attacks. This means narrowing down the analysis to eight specific attacks: DDoS_TCP (DDoS TCP SYN Flood), DDoS_UDP, DDoS_HTTP, DoS_ICMP, MITM (ARP and DNS Spoofing), Password (Password Cracking), SQL injection, and XSS attacks. The distribution of the dataset’s traffic can be seen in the following Figure 4a,b.

To ensure the consistency of analysis, we use the Z-score method for standardisation. Also, to deal with the issue of imbalanced data distribution, we apply the Synthetic Minority Over-sampling Technique (SMOTE) [35]. Lastly, the label encoder is used to convert categorical data into numerical form inspired by [36].

C. Identifying common features:

This stage aims to identify the features that are commonly found in multilayer attacks. This approach starts with iteration over the ‘attack_type’ feature to separate the data based on the nature of the network activity. Following the categorisation, a list of attributes is compiled corresponding to each type of attack. These features have diverse characteristics, such as traffic volume, packet size, and distinct behavioural patterns that help distinguish malicious traffic from normal traffic. We then proceeded to count the frequency of each attribute’s occurrence. This quantification step aids in assessing the distribution of features associated with each attack type. Attributes that appear more frequently suggest a pattern that may be characteristic of a particular form of attack. Out of the initial 64 features, 34 are identified as common features (excluding “label” and “Attack_type”), as shown in Table 1.

**Figure 5:**
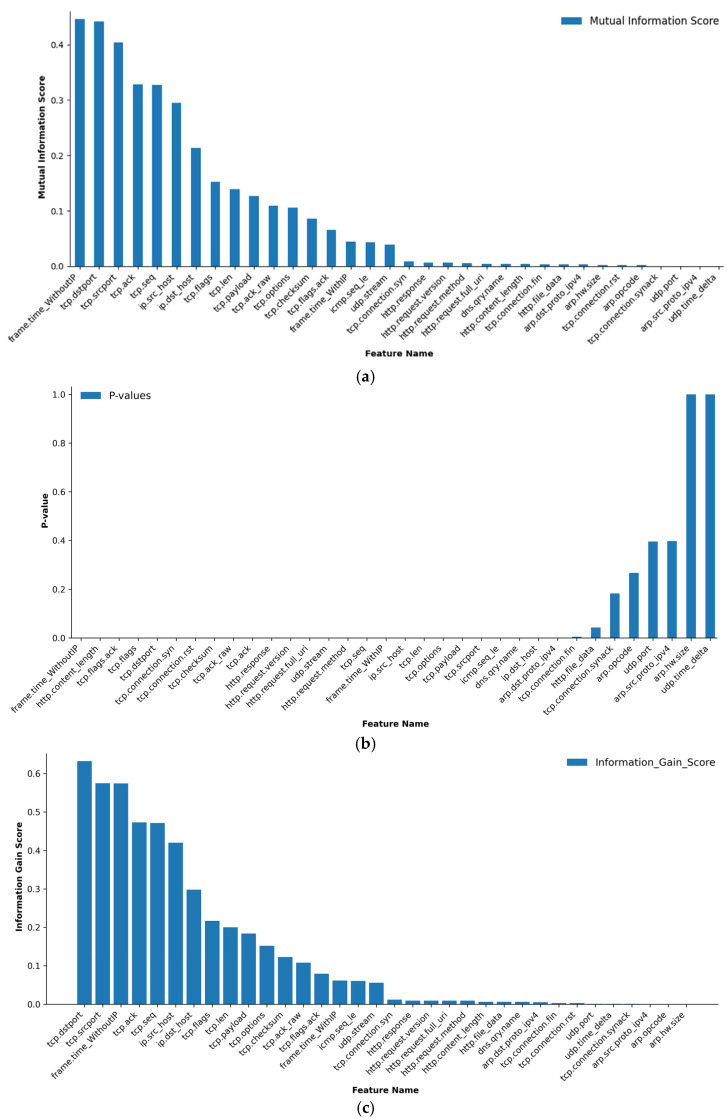

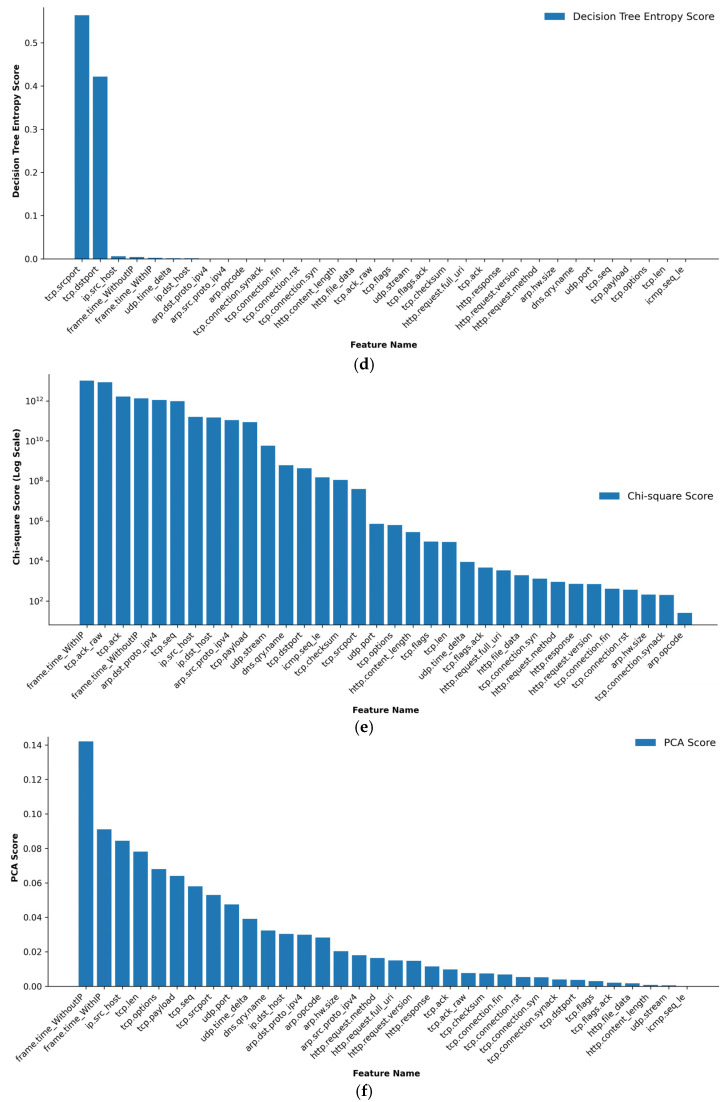

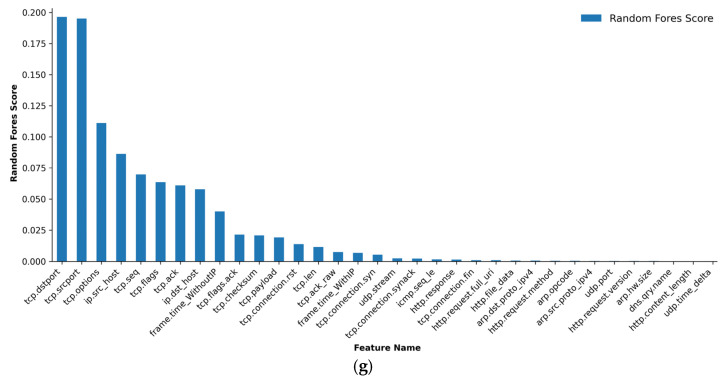
Comparative Analysis of Feature Selection Methods Results for IoT Multilayer Attack Detection. (**a**) Mutual Information Score, (**b**) P-values, (**c**) Information Gain Score, (**d**) Decision Tree Entropy Score, (**e**) Chi-Square Score, (**f**) PCA Score, (**g**) Random Forest Score.

D. Feature Selection Methods:

By identifying and employing the most relevant features, models can be trained more effectively to detect complex patterns associated with multilayer attacks in IoT systems [27]. Six feature selection methods were employed in this research to analyse the strength of the relationship between each feature and the target variable, as illustrated in Figure 5. These methods are Mutual Information (MI), Information Gain (IG), Decision Tree Entropy (DTE), Principal Component Analysis (PCA), Chi-Square (Chi^2^), and Random Forest (RF). These methods were chosen for their effectiveness in handling various data types, including numerical and categorical variables. They allow us to capture both linear and non-linear dependencies between variables and reduce the dimensionality of the feature space, thus improving the efficiency of the classification algorithm [37].

In our analysis, we utilised a permutation test, a statistical method that assesses the significance of the Mutual Information score by comparing it to scores derived from data generated under the null hypothesis (where the features and the target variable are independent) as shown in [38]. Consequently, we calculated *p*-values with the number of permutations set at 1000 for robustness. Out of the 34 considered features, MI identified 26 as significant, highlighting features such as ‘frame.time_WithoutIP’ and ‘tcp.dstport’ due to their strong relationship with the target variable. Conversely, features with *p*-values greater than or equal to 0.05 are considered irrelevant and excluded from further analysis, such as “tcp.connection.synack”, “arp.opcode”, “udp.time_delta”, “arp.src.proto_ipv4”, “udp.port”, “http.file_data”, “tcp.connection.fin”, and “arp.hw.size”.

Using the DTE method, we found 7 out of 34 features to be significant, with “tcp.srcport” and “tcp.dstport” scoring the highest, suggesting their essential role in the classification process. The results of Chi^2^ showed that all features are important, with higher Chi^2^ scores indicating a stronger connection to the target. IG identified 31 out of 34 features as significant, leaving out features such as “arp.hw.size”, “arp.src.proto_ipv4”, and “arp.opcode”, which did not provide valuable information. PCA indicated that 33 out of 34 features are significant, with “frame.time_WithoutIP” and “frame.time_WithIP” being the most influential in the reduced feature space, and “icmp.seq_le“ has been excluded as it does not contribute to classifying the data. Finally, the RF method, which incorporates feature importance as part of its algorithm, successfully identified 27 out of 34 features as significant.

E. Feature Weighting:

Since each feature selection method has its strengths and weaknesses, we decided to incorporate the benefits of the six feature selection methods discussed above and combine their scores. The proposed approach aligns with the ensemble feature selection approach presented in [37]. By combining the scores of various ranking methods, we can assign weight to the features to make the final feature selection more robust and less influenced by any single ranking method.

In our approach, the scores obtained from the six feature selection methods are first normalised using the Min-Max normalisation technique to ensure comparability. The normalised scores from all feature selection methods are then combined to calculate a final score for each feature. The combined score is calculated using the arithmetic mean which averages the normalised scores from the feature selection methods.

**Figure 6:**
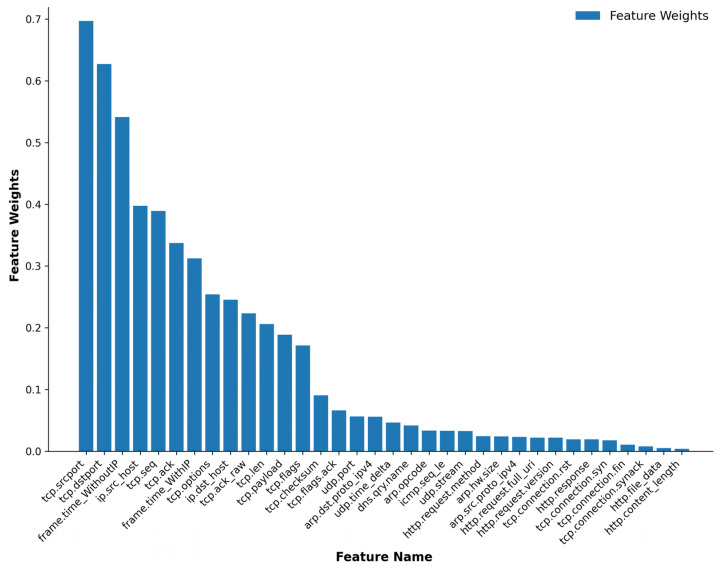
Feature Weights Analysis.

As a result, features are ranked based on their combined scores, with higher scores indicating greater importance. Lastly, each feature is assigned a weight equal to its normalised score as shown in Figure 6. They show that “tcp.srcport” has the highest weight of around 0.7, followed by “tcp.dstport” at around 0.62 and “frame.time_WithoutIP” at 0.54.

**Figure 7:**
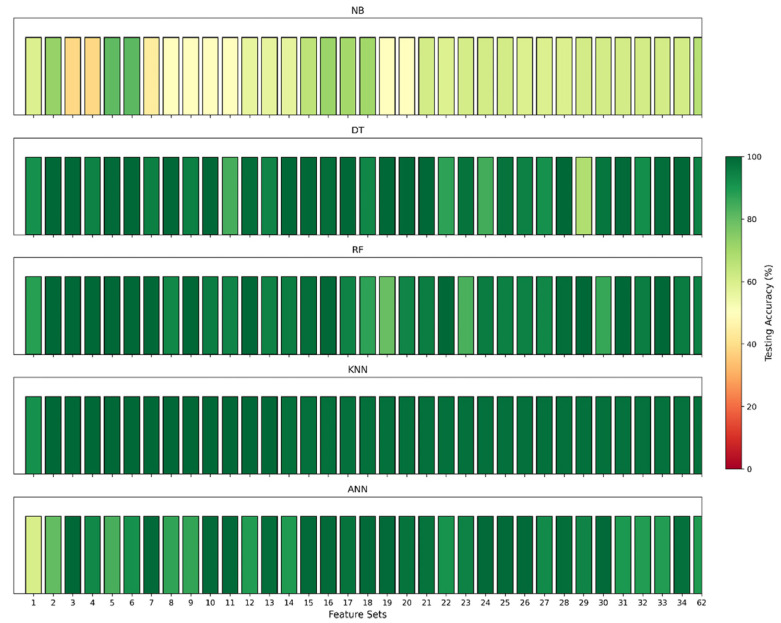
Model accuracies for binary classification across different feature sets.

**Figure 8:**
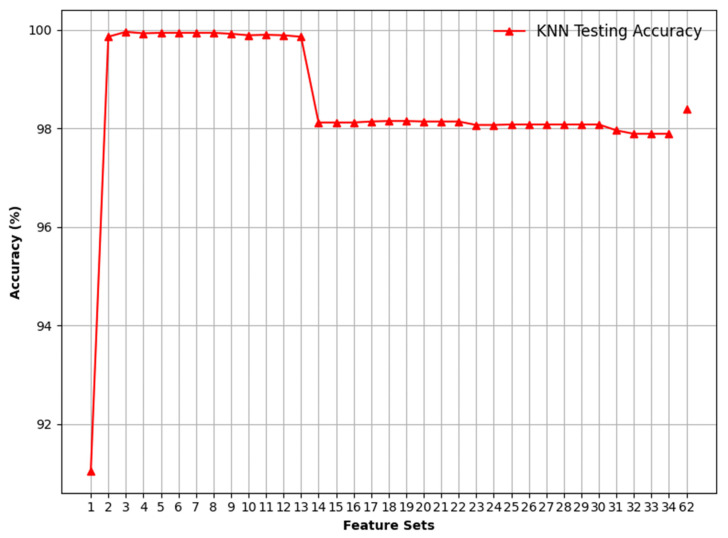
Visualising binary classification using the KNN model.

**Figure 9:**
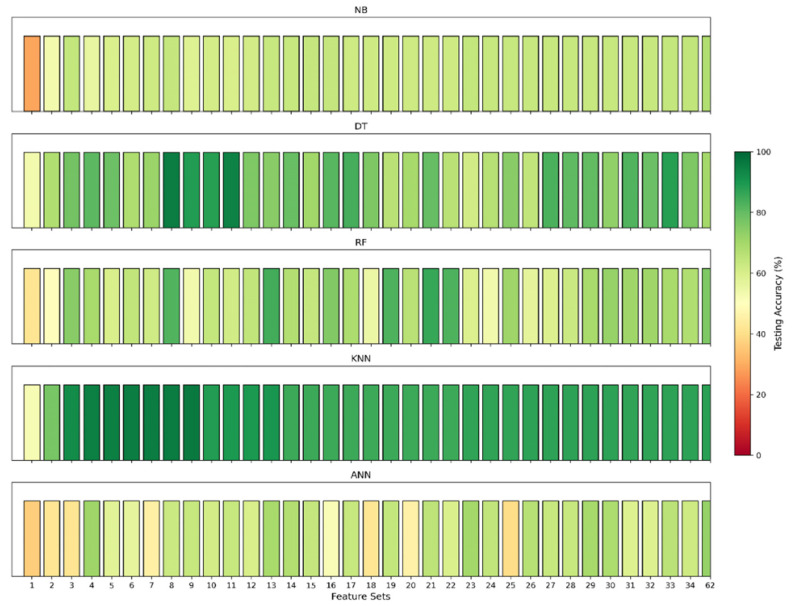
Model accuracies for multiclass classification across different feature sets.

**Figure 10:**
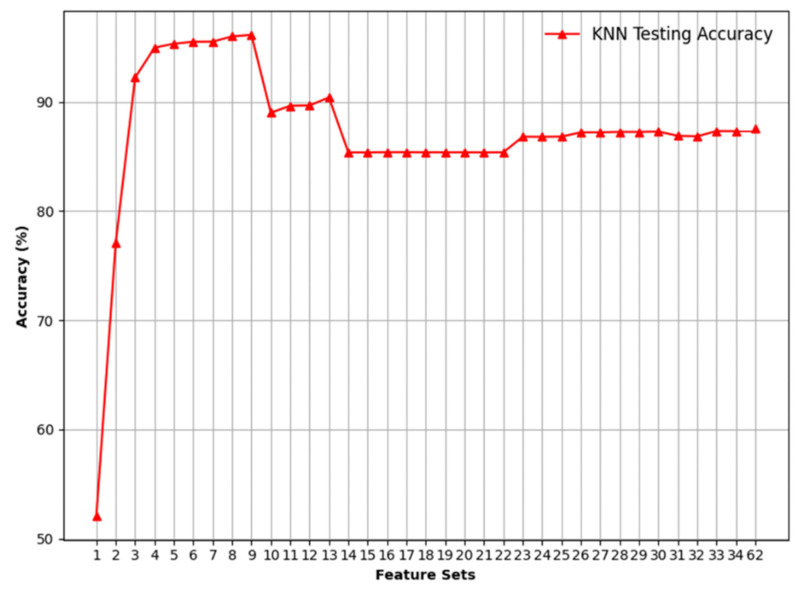
Visualising Multiclass Classification using KNN.

Then, the top-weighted features were added one after another for both binary and multiclass classification to identify the most significant features. Figure 7, Figure 8, Figure 9 and Figure 10 illustrate the model’s testing accuracy using various feature sets, ranging from 1 to 34, and the original 62 features of the Edge-IIoTset dataset.

- Binary classification:

Figure 7 presents the testing accuracies for ML models (DT, KNN, NB, RF, and ANN) for binary classification. Each model’s performance varies across different feature sets, with a colour scale from red to green indicating accuracy levels. Red represents lower accuracy, while green represents higher accuracy. Among these models, the KNN model consistently performs better than the others in this task.

The detailed performance of KNN is shown in Figure 8, which highlights the binary classification results. Initially, the KNN model achieves an accuracy of 91.05% for the first feature set. As additional features are incorporated, the accuracy significantly improves, reaching nearly perfect performance (around 100%) for feature sets 2 to 13. This suggests that these features are highly informative for the binary classification task. However, after feature set 13, there is a noticeable decline in accuracy to approximately 98%. This reduction could be due to the introduction of noise or irrelevant information from the additional features, which can confuse the model and decrease its performance.

- Multiclass Classification:

Figure 9 mirrors the structure of Figure 7 but focuses on multiclass classification. It demonstrates that the KNN model again outperforms the other models, achieving high accuracy. To delve deeper into the KNN model’s performance in multiclass classification, Figure 10 is presented. The testing accuracy starts at a low of 52% with only one feature and increases significantly as more features are added, reaching a peak at the nine-feature set with an accuracy of around 96%. After the nine-feature set, there is a notable drop in accuracy at feature set 10. A slight recovery is seen at the 13-feature set with an accuracy of 90%, followed by another drop. From feature set 14 onward, the accuracy stabilises within the mid-85% range, with minor increases but no return to the peak levels seen with feature sets 9 or 13.

## 5. Results Analysis and Discussion

In this section, our focus was on evaluating the effectiveness of the five classification models in accurately categorising IoT network traffic into normal traffic and multilayer attacks. Each model is optimised through a hyperparameter tuning process known as randomised search, aimed at enhancing their ability to detect multilayer attacks in IoT networks [23].

A. Output of Semi-automated Tool for Identifying Multilayer Attacks

**Figure 11:**
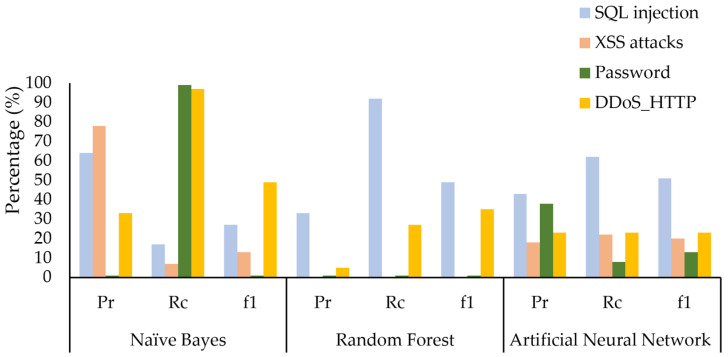
Performance analysis of ML models on 34 common features for multilayer attack identification.

We evaluated the ML models using the 34 common features and all the 62 features identify multilayer attack types. We discovered that with the 34 common features as shown in Figure 11, the RF model failed to detect XSS attacks, as evidenced by zero values in precision, recall, and f-measure. Both NB and ANN demonstrated lower performance in detecting XSS attacks. Additionally, NB demonstrated lower performance in detecting SQL injection attacks and ANN demonstrated lower performance in detecting password attacks.

**Figure 12:**
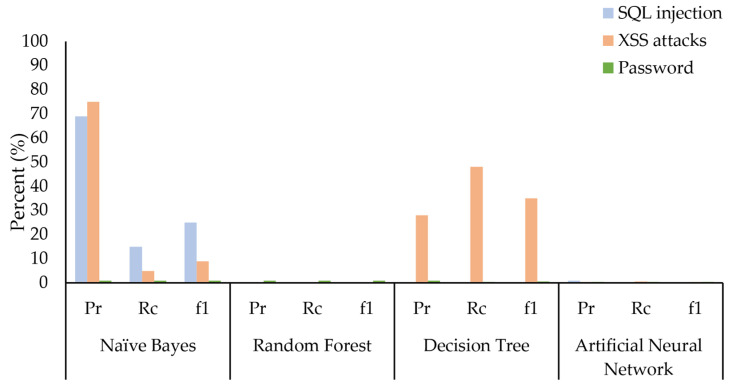
Worst-case analysis of ML models on all 62 features for multilayer attack identification.

In a similar evaluation using all 62 features for classifying multilayer attack types, as shown in Figure 12, we found that RF, DT, and ANN failed to distinguish SQL injection. RF failed to distinguish XSS attacks as well. NB also showed poor performance in detecting XSS attacks and SQL injection. These performance issues with the RF, NB, DT and ANN models in detecting specific multilayer IoT attacks (DDoS_HTTP, XSS, SQL injection, and password attacks) using both the full 62 features and the reduced 34 common features suggest a need for refinement.

Appendix A and Appendix B present the analysis of various machine learning models’ performance using the 13-feature set and the 9-feature set. The 13-feature set includes the following features: “frame.time_WithoutIP”, “frame.time_WithIP”, “ip.src_host”, “ip.dst_host”, “tcp.len”, “tcp.options”, “tcp.payload”, “tcp.seq”, “tcp.srcport”, “tcp.ack”, “tcp.ack_raw”, “tcp.dstport”, and “tcp.flags”. The nine-feature set is a subset of these, comprising “frame.time_WithoutIP”, “frame.time_WithIP”, “ip.src_host”, “ip.dst_host”, “tcp.len”, “tcp.options”, “tcp.payload”, “tcp.seq”, and “tcp.srcport”.

The results show that the models perform well with both feature sets for DDoS attack types (TCP, UDP, HTTP, and ICMP). This indicates that the critical features for detecting these attacks are included in both the 9-feature and 13-feature sets. However, the models do not perform well for password, MITM, XSS, and SQL injection-related attacks, as well as for normal traffic detection.

For the 13-feature set (Appendix A), the NB model displays notably low precision, recall, and F1-score values—2%, 4%, and 3%, respectively—in detecting XSS attacks, indicating a high rate of false positives and false negatives. The testing accuracy is 72%, and the AUC shows a reasonable score in training but experiences a drop in testing from 76% to 68%. This decline could suggest potential overfitting.

In contrast, as demonstrated in (Appendix B), the 9-feature set exhibits significantly poorer performance across several models, such as NB, RF, and ANN, in distinguishing normal traffic, XSS, SQL injection, and password attacks compared to the 13-feature set. While all the metric values for NB in detecting XSS remain very low, they are consistent with those of the 13-feature set. Additionally, for NB, in detecting SQL injection, despite high AUC scores of 97% for both training and testing and an accuracy of 90%, the precision, recall, and f-measure drop to 0. This indicates that the model failed to identify any true positives for SQL injection attacks. For RF, despite high AUC scores for both training and testing and high accuracy in distinguishing normal traffic, XSS, and SQL injection attacks, the model achieved 0% in precision, recall, and f-measure. This suggests that the model failed to identify any true positives for these attacks. The ANN model also exhibits low precision, recall, and f-measure values at 35%, 13%, and 19%, respectively, indicating poor performance in identifying XSS attacks, and 26%, 11%, and 16%, respectively, in identifying password attacks.

Although the 9-feature set is generally sufficient for detecting DDoS (TCP, UDP, HTTP, and ICMP) and MITM-related attacks, it is less effective in detecting normal traffic, password, SQL injection, and XSS attacks compared to the 13-feature set. This highlights the importance of including specific features that are critical for identifying normal traffic and more sophisticated attacks such as password, SQL injection, and XSS. The AUC scores for the 13 features are quite high for both training and testing across all models, suggesting good model performance. However, the drop in AUC from training to testing for NB observed in both feature sets may indicate overfitting, particularly for XSS attacks.

In summary, the application of the SAIDS demonstrates that the 13-feature set is more adept at detecting and identifying multilayer attacks compared to the 9-feature set, 34 common features, and all 62 features.

B. Further Evaluation of ML Models for IoT Multilayer Attack Detection Using 13-Feature Set

When comparing the models in Table 2, KNN stands out as the top performer, achieving perfect scores across all metrics for both normal and multilayer attack detection. Specifically, it achieved precision, recall, F1-score, accuracy, and AUC values ranging from 99% to 100% for both categories, indicating exceptional reliability and performance.

RF, DT, and ANN models also exhibit strong performance, with high values across all metrics ranging from 79% to 100%, making them reliable choices for IoT multilayer attack detection. However, ANN shows slightly lower precision and accuracy for normal traffic at 68% and 83%, respectively. NB, while stable in terms of AUC, shows the lowest performance overall, indicating that it may not be the best choice for this application.

**Figure 13:**
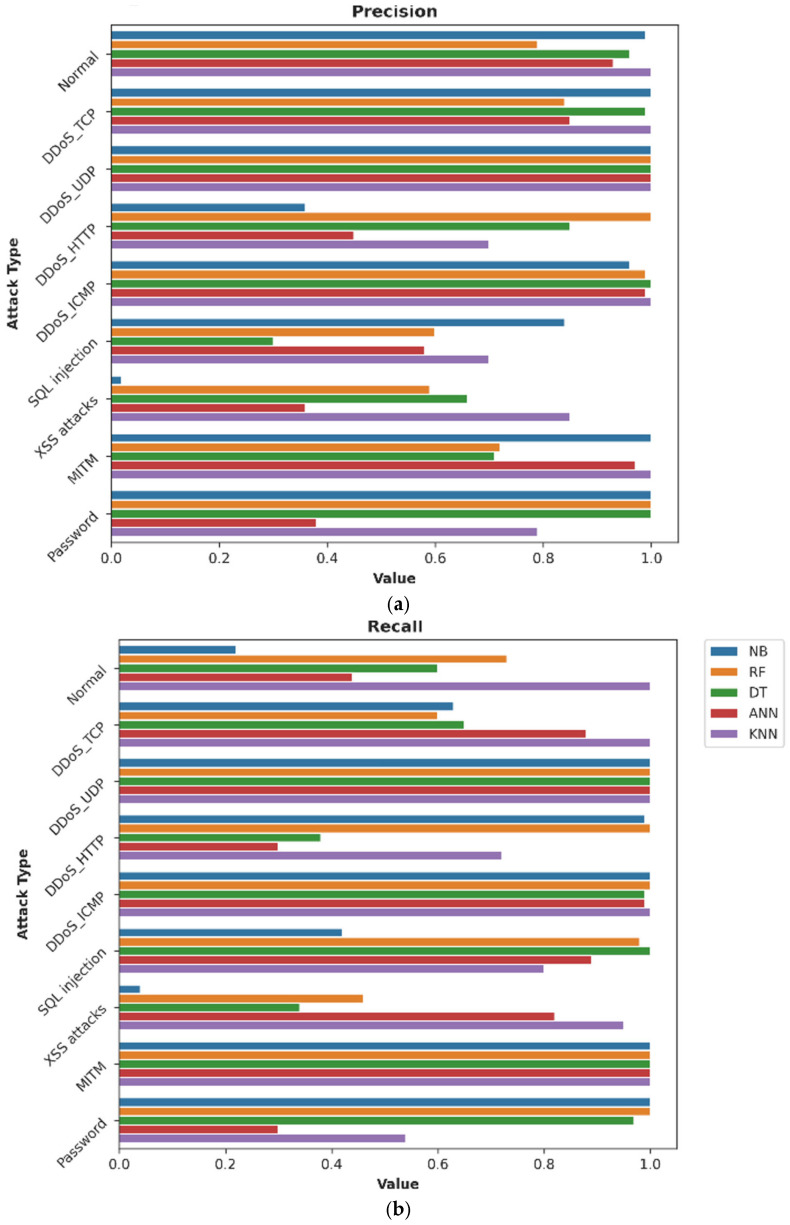

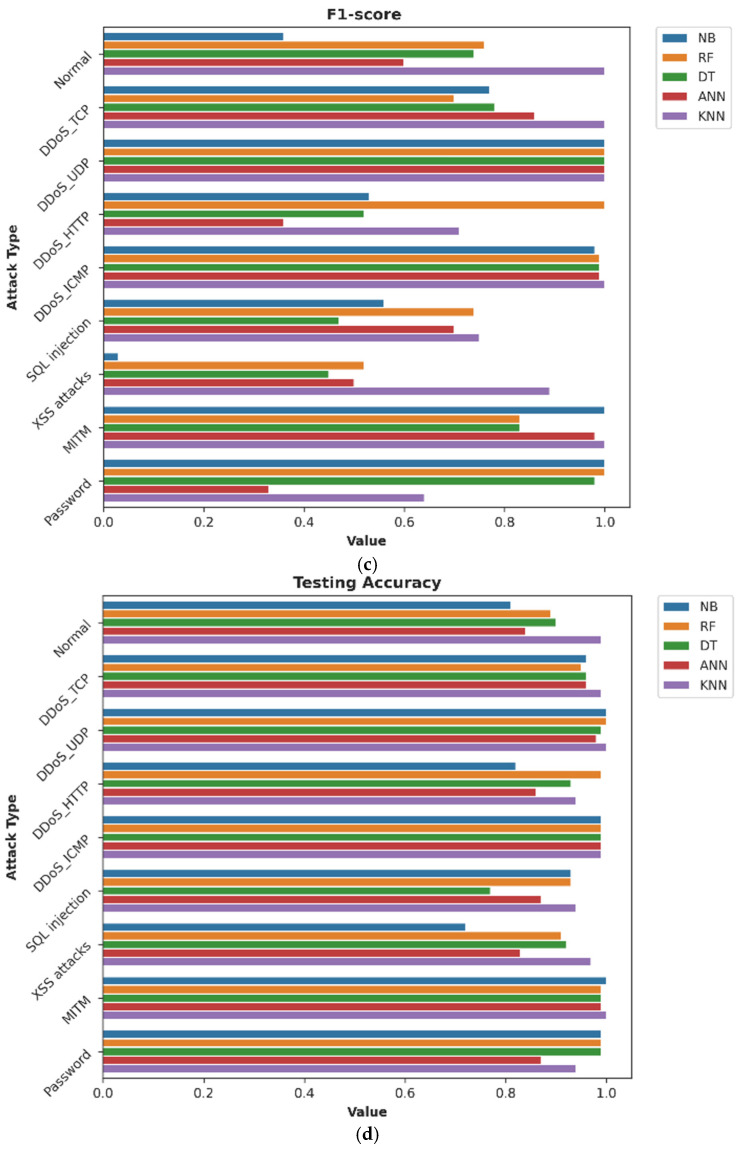

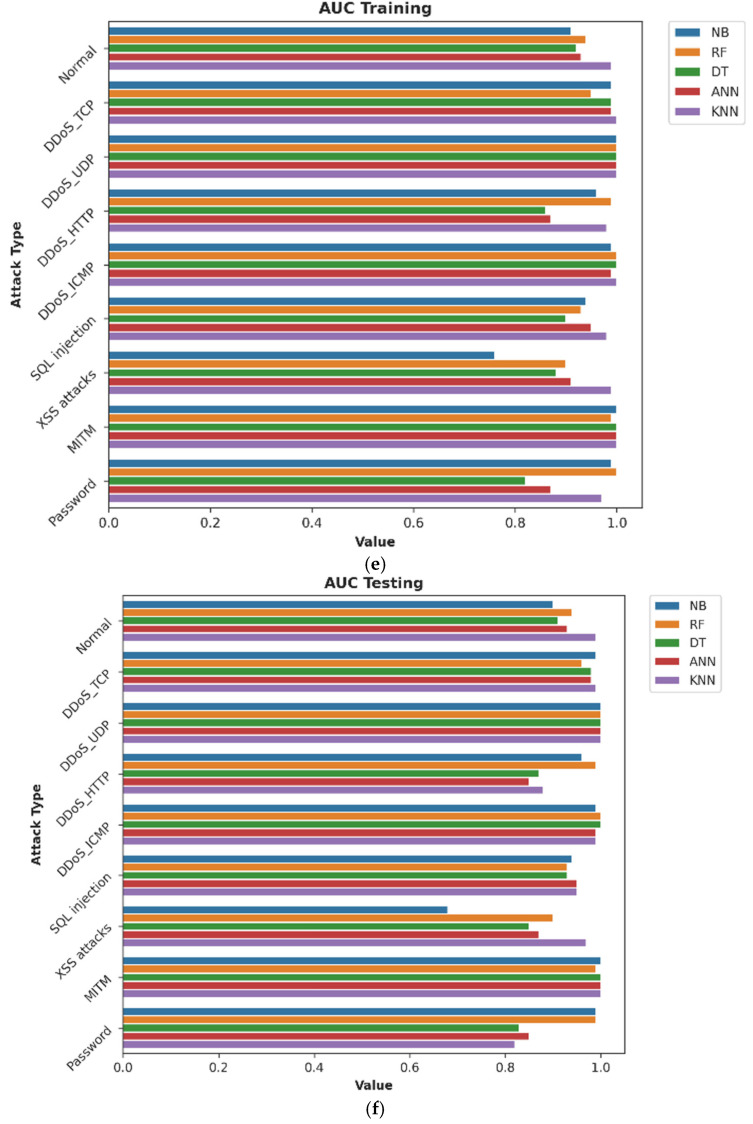
(**a**) precision, (**b**) recall, (**c**) F1-score, (**d**) testing accuracy, (**e**) AUC training and (**f**) testing for 13-feature set.

Figure 13 highlights the key performance indicators for each machine learning model across different attack types. In this figure, KNN and RF generally show the highest precision across most attack types (Figure 13a), with KNN achieving nearly perfect precision for all attack types (ranging from 70% to 100%). NB shows a lower precision of 2% for XSS attacks, suggesting that this area requires significant improvement. Such failings have a significant impact on the practical deployment of the model, resulting in numerous false alerts or missed attacks.

KNN and RF again lead in recall (Figure 13b), indicating their effectiveness in identifying true positive instances of attacks. ANN shows a high recall for most attack types, though it dips for normal traffic, DDoS_HTTP, and password attacks, achieving 44%, 30% and 30%, respectively. NB shows a lower recall of 4% for XSS attacks. KNN consistently achieves high F1-scores (Figure 13c) across all attack types, followed by RF, DT, and ANN. NB shows lower F1-scores for XSS attacks, indicating a balance between precision (Figure 13a), recall (Figure 13b) and F1-scores (Figure 13c) that needs improvement.

All the models demonstrate good testing accuracy across all attack types ranging from 72% to 100% (Figure 13d). KNN outperforms the other models, with an accuracy of 100% in detecting DDoS_UDP and MITM, 99% in detecting normal traffic, DDoS_TCP, and DDoS_ICMP, 97% in detecting XSS, and 94% in detecting DDoS_HTTP, SQL injection, and password attacks.

The AUC values (Figure 13e,f)) for both training and testing are high for KNN, RF, DT, and ANN across all attack types, suggesting strong model performance. NB shows stable AUC but lower values for XSS attacks.

**Figure 14:**
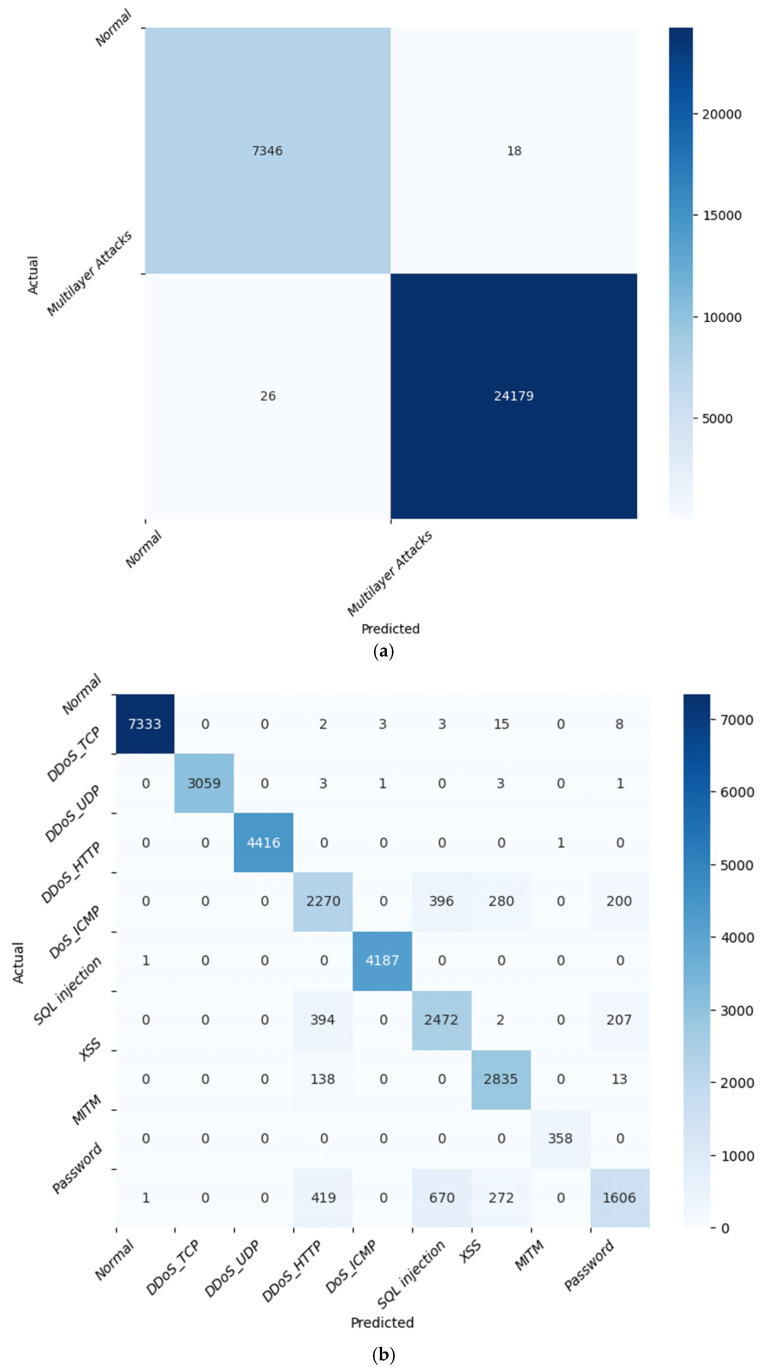
Confusion Matrix using KNN for 13 feature set: (**a**) attack detection, (**b**) attack identification.

Figure 14a displays the KNN model’s accurate classification of normal traffic and multilayer attacks. The confusion matrix indicates that a total of 7364 instances were predicted as ‘Normal’ and 24,179 as ‘Multilayer Attacks’. Only 18 instances were incorrectly labelled as ‘Multilayer Attacks’, and 26 instances were incorrectly classified as ‘Normal’. This means that the model has a high true positive rate, suggesting it is effective at detecting ‘Multilayer Attacks’. The relatively low rates of false positives and false negatives indicate that the model is also capable of correctly identifying ‘Normal’ traffic. Figure 14b relates to multiclass classification, where the KNN model identifies various types of multilayer attacks. It accurately predicted normal traffic (7333 samples), DDoS_TCP (3059 samples), DDoS_UDP (4416 samples), DDoS_HTTP (2270 samples), DoS_ICMP (4187 samples), SQL injection (2472 samples), XSS (2835 samples), MITM (358 samples), and password attacks (1606 samples).

C. Comparative Analysis of IoT Attack Detection Using Edge-IIoTset Dataset

**Figure 15:**
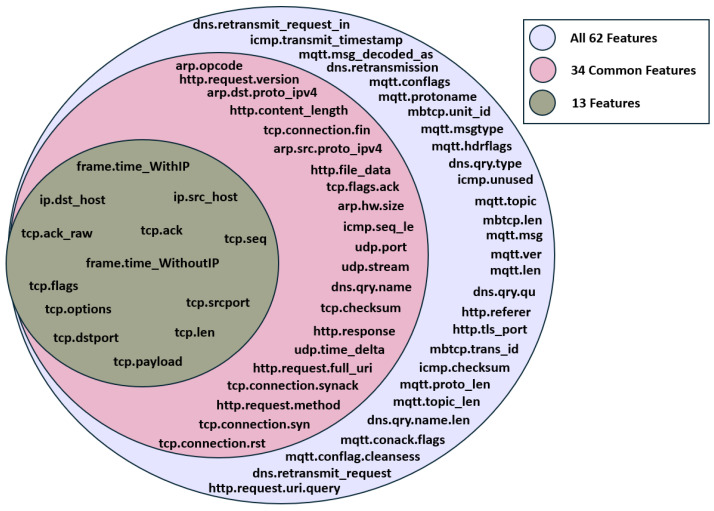
Intersection of all features, 34 common features, and 13 feature sets.

Table 3 presents a comparative analysis of the proposed SAIDS with recent related works [28,29,30,31,32,33,34] that utilised the Edge-IIoTset dataset for IoT attacks detection. The proposed SAIDS stands out by incorporating multilayer IoT attack detection, which is not addressed by the other studies. Additionally, this approach uses a significant 13 features, as illustrated in Figure 15, which is fewer than the features used by most other studies, which range from 20 to 63 features.

Table 4 provides a comprehensive overview of existing datasets used particularly in Intrusion Detection Systems for detecting IoT cyber-attacks. As shown in the table, widely used datasets in network security research, such as KDDCUP 1999, NSL-KDD, UNSW-NB15, CICIDS2017, and CICDDoS2019, are not specific to IoT systems. In contrast, the BoT-IoT, ToN-IoT, Edge-IIoT, and BoTNeT-IoT datasets are specifically designed for IoT systems, containing the unique characteristics of IoT traffic. The BoT-IoT dataset, introduced in 2018, contains 45 features and includes data on DoS and DDoS attacks from multilayer attacks. Similarly, the BoTNeT-IoT dataset, also released in 2018, is limited to only two botnet attacks (Mirai and Gafgyt) and includes just 23 features. On the other hand, the ToN-IoT dataset, introduced in 2020, represents a more comprehensive IoT dataset with 44 features, designed to capture a broad range of IoT traffic and various types of multilayer attacks. Lastly, the Edge-IIoTset dataset, released in 2022, is particularly focused on Industrial IoT (IIoT) environments, providing 61 features that address the complexity of real IoT device traffic and include most of the multilayer attacks.

## 6. Conclusions

In conclusion, this paper addresses a crucial gap in the literature for ML-based multilayer attack detection frameworks, where existing work focuses on specific types of attacks, and utilises outdated datasets, but lacks dynamic, adaptive approaches. Our research introduces the SAIDS, a comprehensive framework designed to detect and identify multilayer attacks. This framework draws upon an established taxonomy of multilayer attacks, integrates efficient feature selection, feature weighting, normalisation, and visualisation, and incorporates human expertise with machine learning algorithms to tackle these attacks using an optimal number of significant features. By utilising the Edge-IIoTset dataset as a case study to implement the SAIDS framework, we identified 13 significant features critical for the detection and classification of multilayer attacks.

The SAIDS framework’s effectiveness is demonstrated by the KNN model’s high accuracy in both binary and multiclass classification, achieving over 94% accuracy in detecting multilayer attacks. Acknowledging the observed limitations, particularly the underperformance of certain models like Naive Bayes in detecting XSS attacks due to its classification approach being less suited to complex attack types, it is evident that our approach could benefit from the adoption of more advanced methodologies. A significant extension to our work would be the incorporation of Continuous Machine Learning (CML) as utilised by [47,48]. Incorporating CML into SAIDS would enable the system to automatically update its understanding of multilayer attack patterns, reducing the likelihood of overfitting seen with static datasets and enhancing the detection of multilayer threats. CML could also lead to the development of more robust feature selection and weighting algorithms that dynamically adjust to the changing landscape of IoT multilayer threats.

Future research directions could also involve deploying the SAIDS in real-life scenarios to validate and refine the system further. This would provide invaluable insights into the system’s real-world performance, revealing unforeseen challenges and enabling the design of solutions to meet practical needs more effectively.

## Figures and Tables

**Table 1:** Thirty-four Common Features.

No.	Feature Name	No.	Feature Name	No.	Feature Name
1	frame.time_WithoutIP	13	arp.dst.proto_ipv4	25	tcp.connection.rst
2	frame.time_WithIP	14	arp.opcode	26	tcp.connection.syn
3	ip.src_host	15	arp.hw.size	27	tcp.connection.synac
4	ip.dst_host	16	arp.src.proto_ipv4	28	tcp.dstport
5	tcp.len	17	http.request.method	29	tcp.flags
6	tcp.options	18	http.request.full_uri	30	tcp.flags.ack
7	tcp.payload	19	http.request.version	31	udp.stream
8	tcp.seq	20	http.response	32	http.file_data
9	tcp.srcport	21	tcp.ack	33	http.content_length
10	udp.port	22	tcp.ack_raw	34	icmp.seq_le
11	udp.time_delta	23	tcp.checksum		
12	dns.qry.name	24	tcp.connection.fin		

**Table 2:** Comparative analysis of IoT multilayer attacks detection using different ML models.

ML Model	Metric	Normal	Multilayer
NB	Precision	0.34	0.97
	Recall	0.51	0.44
	f1-score	0.54	0.61
	Accuracy	0.56	0.56
	AUC Training	0.92	0.92
	AUC Testing	0.92	0.92
RF	Precision	0.83	0.98
	Recall	0.95	0.94
	f1-score	0.88	0.96
	Accuracy	0.94	0.94
	AUC Training	0.98	0.98
	AUC Testing	0.98	0.98
DT	Precision	0.79	1.00
	Recall	1.00	0.92
	f1-score	0.88	0.96
	Accuracy	0.93	0.93
	AUC Training	0.98	0.98
	AUC Testing	0.97	0.97
ANN	Precision	0.68	0.99
	Recall	0.97	0.86
	f1-score	0.80	0.92
	Accuracy	0.83	0.99
	AUC Training	0.99	0.99
	AUC Testing	0.99	0.99
KNN	Precision	1.00	1.00
	Recall	1.00	1.00
	f1-score	1.00	1.00
	Accuracy	0.99	0.99
	AUC Training	0.99	0.99
	AUC Testing	0.99	0.99

**Table 3:** Comparison between proposed model and relevant works on Edge-IIoTset dataset.

Ref.	Models	Multilayer Detection	Features	Feature Selection	Feature Weighting	Hyper-Parameter Opt.	Classify
Keserwani et al. [29]	CatBoost, XGBoos, RF, DT	No	20	Manual	No	No	Binary, Multiclass
Tareq et al. [30]	Inception Time, DenseNet	No	All 63	_	No	Yes	Multiclass
Khacha et al. [31]	CNN-LSTM	No	_	_	No	Yes	Binary, Multiclass
Al Nuaimi et al. [32]	J48, PART, BayesNets, AdaBoos, LogitBoost, ASC	No	All 63	_	No	Yes	Binary, Multiclass
Samin et al. [33]	NB, DT	No	46	Manual	No	No	Multiclass
Ullah et al. [34]	MAGRU	No	31	XGBoost	No	Yes	Multiclass
Ferrag et al. [28]	RF, DT, SVM, KNN, DNN	No	46	Manual	No	Yes	Binary, Multiclass
The proposed method	SVM, DT, KNN, RF, ANN	Yes	13	MI, DTE, IG, RF, Chi^2^	Yes	Yes	Binary, Multiclass

**Table 4:** Summary of datasets used in IoT intrusion detection systems.

Dataset	Reference	Year	IoT Specific	Total Features	Attack Type
KDDCUP 99	Vibhute et al. [39]	1999	No	41	Single
NSL-KDD	Aljawarneh et al. [40]	2009	No	43	Single
UNSW-NB15	Ahmad, Z. et al. [41]	2015	No	49	Single
CICIDS2017	Salman et al. [42]	2017	No	80	Single
BoT-IoT	Peterson et al. [43]	2018	Yes	45	Multilayer
BoTNeT-IoT	[44]	2018	Yes	23	Multilayer
CICDDoS2019	Rehman et al. [45]	2019	No	86	Single
ToN-IoT	Alsaedi et al. [46]	2020	Yes	44	Multilayer
Edge-IIoTset	Ferrag et al. [28]	2022	Yes	61	Multilayer

## Data Availability

Data are contained within the article.

## Abbreviations

ANN	Artificial Neural networks
ARP	Address Resolution Protocol
AUC	Area Under the Curve
CART	Classification and Regression Tree
Chi^2^	Chi-Square
CML	Continuous Machine Learning
CNN	Convolutional Neural Network
DDoS	Distributed Denial of Service Attack
DNN	Deep Neural Networks
DNS	Domain Name System
DoS	Denial of Service Attack
DT	Decision Tree
DTE	Decision Tree Entropy
F1	F1-score
FCBF	Fast-Based-Correlation Feature
FA	Firefly Algorithm
GR	Gain Ratio
GWO	Gray Wolf Optimization
HTTP	Hypertext Transfer Protocol
ICMP	Internet Control Message Protocol
IDC	International Data Corporation
IDS	Intrusion Detection System
IG	Information Gain
IoT	Internet of Things
KNN	K-Nearest Neighbors
LEDEM	Learning-Driven Detection Mitigation
LSTM	Long Short-Term Module
MAGRU	Multi-Head Attention-Based Gated Recurrent Unit
MANET	Mobile Ad Hoc Networks
MI	Mutual Information
MITM	Man-In-The-Middle
ML	Machine Learning
NB	Naïve Bayes
PCA	Principal Component Analysis
Pr	Precision
PSO	Particle Swarm Optimization
R2L	Remote-to-Local
Rc	Recall
RF	Random Forest
RFID	Radio Frequency Identification
SA	Statistical Aggregation
SAIDS	Semi-Automated Intrusion Detection System
SMOTE	Synthetic Minority Oversampling Technique
SVM	Support Vector Machine
SQL	Structured query language
TCP	Transmission Control Protocol
U2R	User-to-Root
UDP	User datagram protocol
XGBoost	Extreme Gradient Boosting
XSS	Cross-Site Scripting

## Appendix A

Analysis of ML models’ performance on 13-feature sets for multilayer attack identification. Yellow highlights indicate worst prediction rates.

**Algorithm**	**Metric**	**Normal**	**DDoS_TCP**	**DDoS_UDP**	**DDoS_HTTP**	**DDoS_ICMP**	**SQL**	**XSS**	**MITM**	**Password**
**NB**	Precision	0.99	1.00	1.00	0.36	0.96	0.84	***0.02***	1.00	1.00
	Rc	0.22	0.63	1.00	0.99	1.00	0.42	***0.04***	1.00	1.00
	f1	0.36	0.77	1.00	0.53	0.98	0.56	***0.03***	1.00	1.00
	Acc.	0.81	0.96	1.00	0.82	0.99	0.93	***0.72***	1.00	0.99
	AUC Training	0.91	0.99	1.00	0.96	0.99	0.94	***0.76***	1.00	0.99
	AUC Testing	0.90	0.99	1.00	0.96	0.99	0.94	***0.68***	1.00	0.99
**RF**	Pr	0.79	0.84	1.00	1.00	0.99	0.60	0.59	0.72	1.00
	Rc	0.73	0.60	1.00	1.00	1.00	0.98	0.46	1.00	1.00
	f1	0.76	0.70	1.00	1.00	0.99	0.74	0.52	0.83	1.00
	Acc.	0.89	0.95	1.00	0.99	0.99	0.93	0.91	0.99	0.99
	AUC Training	0.94	0.95	1.00	0.99	1.00	0.93	0.90	0.99	1.00
	AUC Testing	0.94	0.96	1.00	0.99	1.00	0.93	0.90	0.99	0.99
**DT**	Pr	0.96	0.99	1.00	0.85	1.00	0.30	0.66	0.71	1.00
	Rc	0.60	0.65	1.00	0.38	0.99	1.00	0.34	1.00	0.97
	f1	0.74	0.78	1.00	0.52	0.99	0.47	0.45	0.83	0.98
	Acc.	0.90	0.96	0.99	0.93	0.99	0.77	0.92	0.99	0.99
	AUC Training	0.92	0.99	1.00	0.86	1.00	0.90	0.88	1.00	0.82
	AUC Testing	0.91	0.98	1.00	0.87	1.00	0.93	0.85	1.00	0.83
**ANN**	Pr	0.93	0.85	1.00	0.45	0.99	0.58	0.36	0.97	0.38
	Rc	0.44	0.88	1.00	0.30	0.99	0.89	0.82	1.00	0.30
	f1	0.60	0.86	1.00	0.36	0.99	0.70	0.50	0.98	0.33
	Acc.	0.84	0.96	0.98	0.86	0.99	0.87	0.83	0.99	0.87
	AUC Training	0.93	0.99	1.00	0.87	0.99	0.95	0.91	1.00	0.87
	AUC Testing	0.93	0.98	1.00	0.85	0.99	0.95	0.87	1.00	0.85
**KNN**	Pr	1.00	1.00	1.00	0.70	1.00	0.70	0.85	1.00	0.79
	Rc	1.00	1.00	1.00	0.72	1.00	0.80	0.95	1.00	0.54
	f1	1.00	1.00	1.00	0.71	1.00	0.75	0.89	1.00	0.64
	Acc.	0.99	0.99	1.00	0.94	0.99	0.94	0.97	1.00	0.94
	AUC Training	0.99	1.00	1.00	0.98	1.00	0.98	0.99	1.00	0.97
	AUC Testing	0.99	0.99	1.00	0.88	0.99	0.95	0.97	1.00	0.82

## Appendix B

Analysis of ML models performance on 9-feature sets for multilayer attack identification. Yellow highlights indicate worst predictions.

**Alg**	**Metric**	**Normal**	**DDoS_TCP**	**DDoS_UDP**	**DDoS_HTTP**	**DDoS_ICMP**	**SQL**	**XSS**	**MITM**	**Password**
**NB**	Precision	0.99	1.00	1.00	0.32	0.96	***0.00***	***0.02***	1.00	1.00
	Rc	0.17	0.65	1.00	0.99	1.00	***0.00***	***0.04***	1.00	1.00
	f1	0.29	0.79	1.00	0.48	0.98	***0.00***	***0.03***	1.00	1.00
	Acc.	0.80	0.96	1.00	0.78	0.99	***0.90***	***0.72***	1.00	0.99
	AUC Training	0.92	0.99	1.00	0.99	0.99	***0.97***	***0.76***	1.00	0.99
	AUC Testing	0.92	0.99	1.00	0.99	0.99	***0.97***	***0.68***	1.00	0.99
**RF**	Pr	***0.00***	1.00	1.00	0.19	0.99	***0.00***	***0.00***	0.21	1.00
	Rc	***0.00***	0.60	1.00	1.00	1.00	***0.00***	***0.00***	1.00	1.00
	f1	***0.00***	0.75	1.00	0.32	1.00	***0.00***	***0.00***	0.34	1.00
	Acc.	***0.76***	0.96	1.00	0.58	0.99	***0.90***	***0.90***	0.95	0.99
	AUC Training	***0.78***	0.89	1.00	0.79	1.00	***0.79***	***0.77***	0.99	0.99
	AUC Testing	***0.77***	0.88	1.00	0.76	1.00	***0.76***	***0.73***	0.99	0.99
**DT**	Pr	0.98	1.00	0.99	0.92	1.00	0.67	0.64	1.00	0.93
	Rc	0.92	1.00	1.00	0.58	1.00	1.00	0.95	1.00	0.51
	f1	0.95	1.00	1.00	0.72	1.00	0.80	0.76	1.00	0.66
	Acc.	0.97	1.00	0.99	0.95	1.00	0.95	0.94	1.00	0.95
	AUC Training	0.99	1.00	0.99	0.97	1.00	0.96	0.97	1.00	0.96
	AUC Testing	0.99	1.00	1.00	0.97	1.00	0.97	0.97	1.00	0.97
**ANN**	Pr	0.61	0.40	1.00	0.23	0.95	0.53	***0.35***	0.88	***0.26***
	Rc	0.43	0.69	0.99	0.27	1.00	1.00	***0.13***	1.00	***0.11***
	f1	0.51	0.51	1.00	0.25	0.97	0.70	***0.19***	0.94	***0.16***
	Acc.	0.80	0.87	0.99	0.83	0.99	0.91	***0.89***	0.99	***0.88***
	AUC Training	0.86	0.88	1.00	0.78	0.99	0.95	***0.90***	1.00	***0.83***
	AUC Testing	0.86	0.87	1.00	0.76	0.99	0.90	***0.87***	1.00	***0.80***
**KNN**	Pr	1.00	1.00	1.00	0.83	0.99	0.87	0.91	1.00	1.00
	Rc	1.00	0.99	1.00	0.81	1.00	0.87	0.97	1.00	0.98
	f1	1.00	0.99	1.00	0.82	1.00	0.87	0.94	1.00	0.99
	Acc.	0.99	0.99	1.00	0.96	0.99	0.97	0.98	1.00	0.99
	AUC Training	1.00	1.00	1.00	0.99	1.00	0.99	0.99	1.00	1.00
	AUC Testing	0.99	0.99	1.00	0.96	0.99	0.98	0.99	1.00	0.99

## References

[1] Future of Industry Ecosystems: Shared Data and Insights. https://blogs.idc.com/2021/01/06/future-of-industry-ecosystems-shared-data-and-insights/.

[2] NCSC For Startups: Challenges. https://www.ncsc.gov.uk/section/ncsc-for-startups/current-challenges.

[3] X-Force Threat Intelligence Index 2022. https://www.ibm.com/downloads/cas/ADLMYLAZ.

[4] Organisational Use of Enterprise Connected Devices. https://www.ncsc.gov.uk/report/organisational-use-of-enterprise-connected-devices.

[5] Khanam S., Ahmedy I.B., Idna Idris M.Y., Jaward M.H., Bin Md Sabri A.Q. (2020). A Survey of Security Challenges, Attacks Taxonomy and Advanced Countermeasures in the Internet of Things. IEEE Access.

[6] Mitrokotsa A., Rieback M.R., Tanenbaum A.S. (2010). Classifying RFID attacks and defenses. Inf. Syst. Front..

[7] Atlam H.F., Wills G.B. (2019). IoT Security, Privacy, Safety and Ethics. Digital Twin Technologies and Smart Cities.

[8] Ahmad R., Alsmadi I. (2021). Machine learning approaches to IoT security: A systematic literature review. Internet Things.

[9] Bansal D., Sofat S. Use of cross layer interactions for detecting denial of service attacks in WMN. Proceedings of the 2010 14th International Telecommunications Network Strategy and Planning Symposium (NETWORKS).

[10] Bansal D., Sofat S., Kumar P. Distributed cross layer approach for detecting multilayer attacks in wireless multi-hop networks. Proceedings of the 2011 IEEE Symposium on Computers & Informatics.

[11] Sodagudi S., Rao D.K.R. (2014). Behavior based Anomaly detection technique to identify Multilayer attacks. Int. J. Adv. Res. Comput. Sci. Manag. Stud..

[12] Mahale V.V., Pareek N.P., Uttarwar V.U. (2017). Alleviation of DDoS attack using advance technique. Proceedings of the 2017 International Conference on Innovative Mechanisms for Industry Applications (ICIMIA).

[13] Mythili B., Seetha R. (2021). Accurate Detection of Multi-layer Packet Dropping Attacks Using Distributed Mobile Agents in MANET. J. Phys. Conf. Ser..

[14] Chen Y., Sheu J., Kuo Y., Van Cuong N. Design and Implementation of IoT DDoS Attacks Detection System based on Machine Learning. Proceedings of the 2020 European Conference on Networks and Communications (EuCNC).

[15] Ravi N., Shalinie S.M. (2020). Learning-Driven Detection and Mitigation of DDoS Attack in IoT via SDN-Cloud Architecture. IEEE Internet Things J..

[16] Chkirbene Z., Eltanbouly S., Bashendy M., AlNaimi N., Erbad A. Hybrid Machine Learning for Network Anomaly Intrusion Detection. Proceedings of the 2020 IEEE International Conference on Informatics, IoT, and Enabling Technologies (ICIoT).

[17] Bagaa M., Taleb T., Bernabe J.B., Skarmeta A. (2020). A Machine Learning Security Framework for Iot Systems. IEEE Access.

[18] Shafiq M., Tian Z., Bashir A.K., Du X., Guizani M. (2020). IoT malicious traffic identification using wrapper-based feature selection mechanisms. Comput. Secur..

[19] Nimbalkar P., Kshirsagar D. (2021). Feature selection for intrusion detection system in Internet-of-Things (IoT). ICT Express.

[20] Su J., He S., Wu Y. (2022). Features selection and prediction for IoT attacks. High-Confid. Comput..

[21] Albulayhi K., Abu Al-Haija Q., Alsuhibany S.A., Jillepalli A.A., Ashrafuzzaman M., Sheldon F.T. (2022). IoT Intrusion Detection Using Machine Learning with a Novel High Performing Feature Selection Method. Appl. Sci..

[22] Sujatha G., Ayyannan M., Priya S.G., Arun V., Arularasan A.N., Kumar M.J. Hybrid Optimization Algorithm to Mitigate Phishing URL Attacks in Smart Cities. Proceedings of the 2023 3rd International Conference on Innovative Practices in Technology and Management (ICIPTM).

[23] Swathi G., Shwetha M., Potluri P., Murthy Raju K., Kumar Y., Rajchandar K. (2023). Smart Cities Hybridized to Prevent Phishing URL Attacks. Proceedings of the 2023 Second International Conference on Electronics and Renewable Systems (ICEARS).

[24] Khan H.U., Sohail M., Nazir S. (2022). Features-based IoT Security Authentication Framework using Statistical Aggregation, Entropy, and MOORA Approaches. IEEE Access.

[25] Subramani S., Selvi M. (2023). Multi-objective PSO based feature selection for intrusion detection in IoT based wireless sensor networks. Optik.

[26] Al Sukhni B., Manna K.S., Dave M.J., Zhang L. (2022). Investigating the Security Issues of Multi-layer IoT Attacks Using Machine Learning Techniques. Proceedings of the 2022 Human-Centered Cognitive Systems (HCCS).

[27] Al Sukhni B., Manna K.S., Dave M.J., Zhang L. (2023). Exploring Optimal Set of Features in Machine Learning for Improving IoT Multilayer Security. Proceedings of the 2023 IEEE 9th World Forum on Internet of Things (WF-IoT).

[28] Ferrag M.A., Friha O., Hamouda D., Maglaras L., Janicke H. (2022). Edge-IIoTset: A New Comprehensive Realistic Cyber Security Dataset of IoT and IIoT Applications for Centralized and Federated Learning. IEEE Access.

[29] Keserwani K., Aggarwal A., Chauhan A. (2023). Attack detection in industrial IoT using novel ensemble techniques. Proceedings of the 2023 2nd International Conference on Vision Towards Emerging Trends in Communication and Networking Technologies (ViTECoN).

[30] Tareq I., Elbagoury B.M., El-Regaily S., El-Horbaty E.M. (2022). Analysis of ToN-IoT, UNW-NB15, and Edge-IIoT Datasets Using DL in Cybersecurity for IoT. Appl. Sci..

[31] Khacha A., Saadouni R., Harbi Y., Aliouat Z. (2022). Hybrid Deep Learning-based Intrusion Detection System for Industrial Internet of Things. Proceedings of the 2022 5th International Symposium on Informatics and its Applications (ISIA).

[32] Al Nuaimi T., Al Zaabi S., Alyilieli M., AlMaskari M., Alblooshi S., Alhabsi F., Yusof M.F.B., Al Badawi A. (2023). A comparative evaluation of intrusion detection systems on the edge-IIoT-2022 dataset. Intell. Syst. Appl..

[33] Samin O.B., Algeelani N.A.A., Bathich A., Qadus A., Amin A. (2023). Malicious Agricultural IoT Traffic Detection and Classification: A Comparative Study of ML Classifiers. J. Adv. Inf. Technol..

[34] Ullah S., Boulila W., Koubaa A., Ahmad J. (2023). MAGRU-IDS: A Multi-Head Attention-based Gated Recurrent Unit for Intrusion Detection in IIoT Networks. IEEE Access.

[35] Maghrabi L. (2024). Automated Network Intrusion Detection for Internet of Things: Security Enhancements. IEEE Access.

[36] Rashid M.M., Khan S.U., Eusufzai F., Redwan M.A., Sabuj S.R., Elsharief M. (2023). A Federated Learning-Based Approach for Improving Intrusion Detection in Industrial Internet of Things Networks. Network.

[37] Göcs L., Johanyák Z.C. (2023). Feature Selection with Weighted Ensemble Ranking for Improved Classification Performance on the CSE-CIC-IDS2018 Dataset. Computers.

[38] François D., Wertz V., Verleysen M. The permutation test for feature selection by mutual information. Proceedings of the European Symposium on Artificial Neural Networks.

[39] Vibhute A.D., Patil C.H., Mane A.V., Kale K.V. (2024). Towards Detection of Network Anomalies using Machine Learning Algorithms on the NSL-KDD Benchmark Datasets. Procedia Comput. Sci..

[40] Aljawarneh S., Aldwairi M., Yassein M.B. (2018). Anomaly-based intrusion detection system through feature selection analysis and building hybrid efficient model. J. Comput. Sci..

[41] Ahmad Z., Khan A.S., Cheah W.S., Abdullah J.B., Ahmad F. (2021). Network intrusion detection system: A systematic study of machine learning and deep learning approaches. Trans. Emerg. Telecommun. Technol..

[42] Salman O., Elhajj I.H., Chehab A., Kayssi A. (2022). A machine learning based framework for IoT device identification and abnormal traffic detection. Trans. Emerg. Telecommun. Technol..

[43] Peterson J.M., Leevy J.L., Khoshgoftaar T.M. (2021). A Review and Analysis of the Bot-IoT Dataset. Proceedings of the 2021 IEEE International Conference on Service-Oriented System Engineering (SOSE).

[44] Belkacem S. (2024). IoT-Botnet Detection Using Deep Learning Techniques. Proceedings of the International Conference on Information Technology and Applications (ICITA 2022).

[45] Rehman S.u., Khaliq M., Imtiaz S.I., Rasool A., Shafiq M., Javed A.R., Jalil Z., Bashir A.K. (2021). DIDDOS: An approach for detection and identification of Distributed Denial of Service (DDoS) cyberattacks using Gated Recurrent Units (GRU). Future Gener. Comput. Syst..

[46] Alsaedi A., Moustafa N., Tari Z., Mahmood A., Anwar A. (2020). TON_IoT Telemetry Dataset: A New Generation Dataset of IoT and IIoT for Data-Driven Intrusion Detection Systems. IEEE Access.

[47] Ariyadasa S., Fernando S., Fernando S. (2024). SmartiPhish: A reinforcement learning-based intelligent anti-phishing solution to detect spoofed website attacks. Int. J. Inf. Secur..

[48] Seetha A., Chouhan S.S., Pilli E.S., Raychoudhury V. (2023). D i E vD: Disruptive Event Detection from Dynamic Datastreams using Continual Machine Learning: A Case Study with Twitter. IEEE Trans. Emerg. Top. Comput..

